# Study on liver histopathology of chronic HBV infected patients with different normal ALT values

**DOI:** 10.3389/fimmu.2022.1069752

**Published:** 2022-11-22

**Authors:** Zhan Zeng, Hongxiao Hao, Xiaoyue Bi, Yanjie Lin, Liu Yang, Shiyu Wang, Ge Shen, Min Chang, Tingting Jiang, Wen Deng, Huihui Lu, Fangfang Sun, Yao Lu, Yuanjiao Gao, Ruyu Liu, Mengjiao Xu, Xiaoxue Chen, Leiping Hu, Lu Zhang, Minghui Li, Yao Xie

**Affiliations:** ^1^ Department of Hepatology Division 2, Beijing Ditan Hospital, Capital Medical University, Beijing, China; ^2^ Department of Infectious Diseases, Peking University First Hospital, Beijing, China; ^3^ Department of Hepatology Division 2, Peking University Ditan Teaching Hospital, Beijing, China

**Keywords:** chronic HBV-infection, ALT value, HBV therapy, liver histopathology, clinical indicators

## Abstract

**Aims:**

Comparison of liver histopathological findings to explore the occurrence of liver inflammation in patients with chronic hepatitis B (CHB) under different alanine aminotransferase (ALT) normal values.

**Methods:**

The patients who were diagnosed as chronic hepatitis B virus (HBV) infection by liver histopathology at the Department of Pathology, Beijing Ditan Hospital due to clinical difficulty in defining the degree of liver inflammation or fibrosis were retrospectively enrolled from May 2008 to November 2020. Study of the incidence of significant hepatic histopathology in enrolled patients according to different ALT normal values. Using logistic regression to investigate the relevant factors of significant hepatic histopathology.

**Results:**

A total of 1474 patients were enrolled, 56.20% of the patients were male, and the overall patients’ age was 36.80 ± 10.60 years. 39.00% of patients had liver inflammation grade G > 1, 34.70% liver fibrosis stage S > 1, and 48.17% patients had significant hepatic histopathology (G > 1 and/or S > 1). Among patients with normal ALT values, 36.40% and 40.40% had significant hepatic histopathology by American Association for the Study of Liver Diseases (AASLD) criteria and Chinese guideline criteria, respectively, but the difference was not statistically significant (*χ^2 =^* 3.38, *P* =0.066). In contrast, among patients with abnormal ALT values, 58.90% and 62.20% of patients had significant hepatic histopathology by AASLD criteria and Chinese guideline criteria, respectively, with no significant difference (*χ^2 =^* 2.28, *P* =0.131). ALT (*P <*0.001, OR=1.019), hepatitis B surface antigen (HBsAg) (*P <*0.001, OR=0.665) and hepatitis B e antigen (HBeAg) status (*P <*0.001, OR=2.238) were relevant factors in the occurrence of significant hepatic histopathology. ALT was positively corelated with grade of inflammation G (r =0.194, *P <*0.001) and negatively correlated with liver fibrosis stage S (r =-0.066, *P* =0.021).

**Conclusions:**

Our study found no statistically significant differences in the presence of significant hepatic histopathology under the two ALT criteria. ALT, HBsAg and HBeAg status were related to the occurrence of significant hepatic histopathology.

## Introduction

Since the widespread application of the recombinant hepatitis B vaccine in China in 1992, the prevalence of HBV among the Chinese population has declined year by year. The prevalence of HBsAg in the serological survey of HBV in China in 2006 and 2014 decreased by 46% and 52%, respectively, compared with 1992 (1), and the prevalence of children under the age of 5 decreased by 97% (1). With the further application of vaccines, the chronic infection rate of HBV in the Chinese population will further decline. Nevertheless, there are still about 70 million chronic HBV-infected people in China, 20 to 30 million of which are CHB patients requiring treatment, and about 1 million people are newly diagnosed as hepatitis patients every year (2).

The typical natural history of chronic HBV infection includes HBeAg positive chronic infection, HBeAg positive chronic hepatitis, HBeAg negative chronic infection, HBeAg negative chronic hepatitis, and HBsAg negative stage (2, 3). The key to whether patients with chronic HBV infection need to start antiviral treatment is whether hepatitis occurs. If there is inflammation, it needs to be treated. At present, in the latest prevention and treatment guidelines for CHB in China, indicators such as ALT, hepatitis B virus deoxyribonucleic acid (HBV DNA), HBsAg, HBeAg, and liver pathology are used to assess the infection stage of the infected people. Based on the diagnosis and treatment guidelines for CHB updated in 2019, Chinese scholars put forward the expert opinion on expanding the antiviral treatment of CHB (4). The opinion pointed out that the screening of HBsAg in the population should be expanded and highly sensitive HBV DNA detection methods should be used. Lowering the ALT threshold for CHB patients to start treatment, and actively treating patients who are at risk of disease progression or in the gray area (4). The purpose of our study is to retrospectively collect the biochemical indicators and liver pathology results of patients who were chronic infected by HBV, compare the accuracy of ALT normal value under different definitions of CHB guidelines (2, 3, 5, 6) in judging whether there are significant hepatic histopathology in patients with chronic HBV infection, and discuss the necessity of lowering the ALT threshold of patients with CHB for antiviral treatment.

## Methods and materials

### Patients

Patients with chronic HBV infection who had undergone liver biopsy in Beijing Ditan Hospital from May 2008 to November 2020 were enrolled. Demographic data, antiviral treatment information, HBV DNA load, and serological test results, as well as the examination results of liver function, renal function, blood routine and coagulation function indicators were collected. According to the inclusion and exclusion conditions, patients were enrolled and statistically analyzed.

Inclusion criteria: 1) 18-65 years old; 2) The duration of HBsAg positive was more than 6 months; 3) Having not received HBV antiviral treatment; 4) The diagnosis result of liver puncture pathology was clear; 5) Having the complete results of liver function, coagulation function, blood routine test, viral load, and serological index within 2 weeks before or after the biopsy.

Exclusion criteria: 1) Exclude other types of viral hepatitis (including hepatitis A, hepatitis C, and hepatitis E), alcoholic liver disease, non-alcoholic fatty liver disease, drug-related liver injury, autoimmune liver injury, cirrhosis, liver cancer, and other liver diseases; 2) Co-infection with other viruses (such as human immunodeficiency virus, cytomegalovirus, etc.); 3) Patients’ biopsy results didn’t suggest a definite inflammatory grade or fibrosis stage; 4) Those with heart and kidney dysfunction.

The patients are chronic HBV-infected people who are difficult to judge whether there is significant hepatic histopathological change through clinical indicators, and carry out liver puncture examinations.

### Study parameters

Aspartate aminotransferase (AST), ALT, albumin (ALB), total bilirubin (TBIL), cholinesterase (CHE), platelet (PLT), prothrombin time activity (PTA), international normalized ratio (INR), HBsAg, HBeAg, HBV DNA.

The results of liver pathological examination included grade of inflammation G0-G4, and liver fibrosis stage S0-S4. Using Scheuer scoring system.

In this study, G > 1 means that the liver has significant inflammation, and G ≤ 1 means that the liver has no significant inflammation. S > 1 is defined as significant fibrosis of the liver, S0 as no fibrosis, and 0 < S ≤ 1 as slight fibrosis. G ≤ 1 and S ≤ 1 is defined as no significant hepatic histopathology, G > 1 and/or S > 1 as significant hepatic histopathology.

### Study content

Patients were divided into normal ALT group and abnormal ALT group according to the standard of normal ALT (≤ 40 U/L) in 2019 Chinese Hepatitis B Treatment Guidelines, 2019 European Association for the Study of the Liver (EASL) Guidelines, and 2016 Asian Pacific Association for the Study of the Liver (APASL) Guidelines.

According to the guidelines of the American Association for the Study of Liver Diseases (AASLD) for chronic hepatitis B, male patients with ALT ≤ 35 U/L and female patients with ALT ≤ 25 U/L were assigned to normal ALT group, and the rest were classified as abnormal ALT group.

Considering the liver pathology, the incidence of significant hepatic histopathology in normal and abnormal patients was evaluated to compare the difference under the two ALT standards, and the impact of different ALT thresholds on the accuracy of diagnosing significant hepatic histopathology in patients with chronic hepatitis B was studied.

ALT, HBsAg, HBeAg status and HBV DNA load were used as independent variables for univariate regression analysis for the occurrence of significant hepatic histopathology, respectively. Those with statistically significant differences were then selected for multivariate regression analysis. The cut-off values of the factors associated with the occurrence of significant hepatic histopathology were calculated separately.

Finally, patients with significant hepatic histopathology were selected and the correlation between ALT and G, S in these patients were analyzed, respectively.

### Statistical analysis

Describing counting data with mean ± standard deviation (normal data) or quartile (non-normal data), describing classified data with percentage. Comparing the classified data with chi-square test, and analyzing the relavant factors in the occurrence of significant hepatic histopathology with univariate binary logistic regression and receiver operating characteristic curve (ROC). Kendall’s tau-b correlation analysis was used in categorical data. All data were analyzed with IBM SPSS 25.0, and graphed with Graphpad Prism 8.0 and Microsoft Excel 2019. The significance of all tests shall be subject to bilateral tests. If *P <*0.05, it means there is statistical significance.

## Results

### Patients

**Figure 1:**
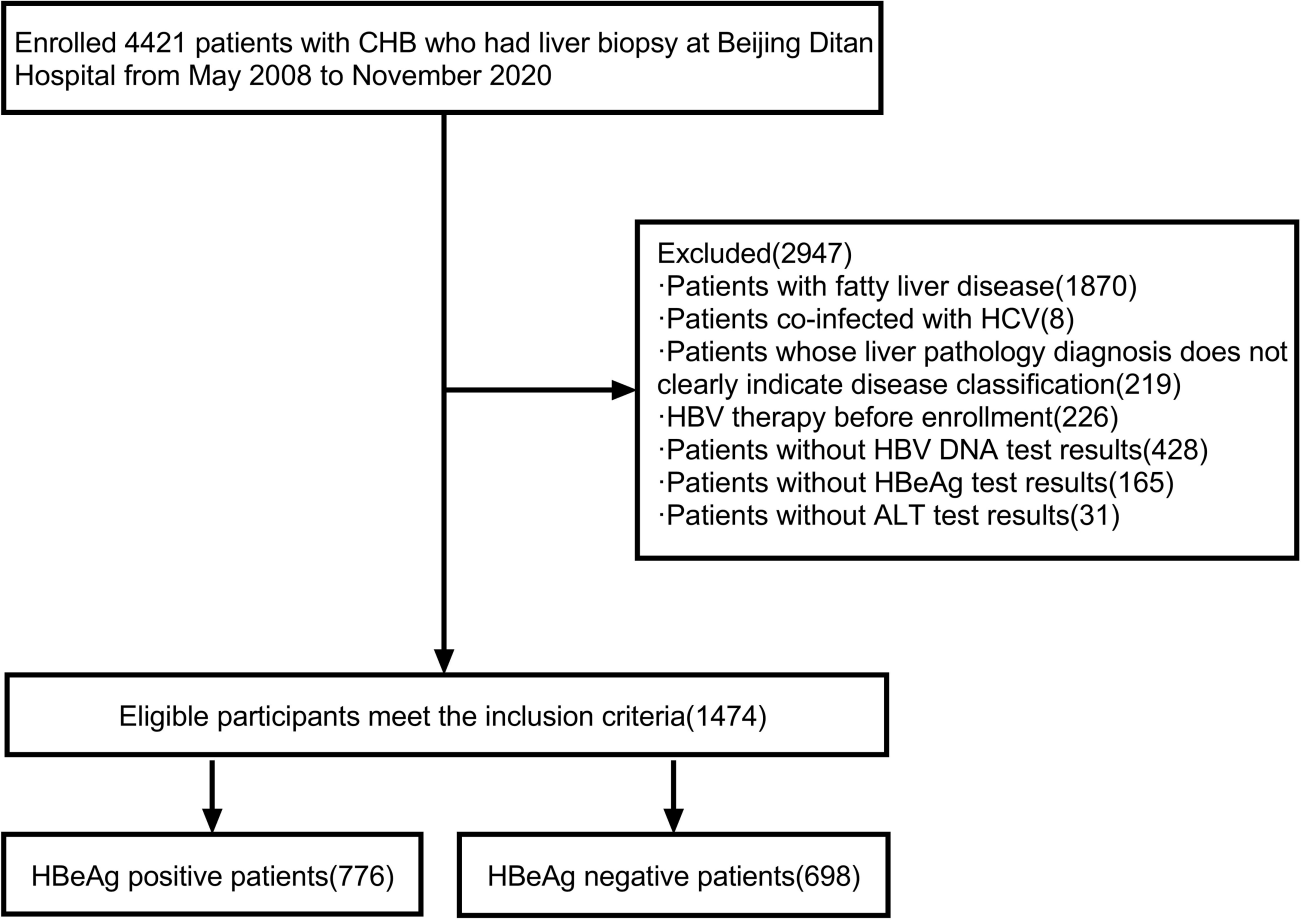
Flow chart of the screening process of the research patients. CHB, chronic hepatitis B; HCV, hepatitis C virus; HBV DNA, hepatitis B virus deoxyribonucleic acid; HBV, hepatitis B virus; ALT, alanine aminotransferase; HBeAg, hepatitis B e antigen.

There were 4421 patients with definite liver histopathological diagnoses. Demographic data, HBV virus content, serological indicators, clinical biochemical, and blood routine indicators were collected. 1870 patients with fatty liver, 8 patients with chronic hepatitis C, 219 patients with no clear indication of disease grade by the pathological diagnosis of liver, 226 patients undergoing HBV therapy at the time of enrollment, 428 patients missing HBV DNA detection results at the time of pathological examination of liver tissue, 165 patients missing HBeAg results, and 31 patients missing ALT results were excluded, Finally, 1474 patients with definite liver histopathological diagnosis, completing virus content and serological, liver function indicators and peripheral blood indicators, were enrolled (**Figure 1**).

Among all patients, 56.20% were male, 36.8 ± 10.6 years old, and 48.17% had significant hepatic histopathology. Patients with significant hepatic histopathology had higher percentage of HBeAg positive patients (59.00% vs 46.70%, *χ* ^2 =^ 22.30, *P <*0.001), higher age (38.00 ± 10.80 vs 35.70 ± 10.20 years old, t=-4.13, *P <*0.001), higher ALT levels [38.2 (25.7, 58.1) vs 26.8 (18.9, 40.7) U/L, z=-10.28, *P <*0.001], and higher AST levels [32.8 (24.4, 48.9) vs 24 (19.3, 33.5) U/L, z=-11.69, *P <*0.001]. Patients with significant hepatic histopathology had lower HBsAg levels (3.20 ± 1.18 vs 3.38 ± 1.25 log10 IU/ml, t=2.25, *P* =0.025), lower ALB levels (44.10 ± 4.49 vs 46.20 ± 3.58 g/L, t=9.73, *P <*0.001), lower PLT counts (166.00 ± 57.90 vs 199.00 ± 51.20 109/L, t=11.52, *P <*0.001), and lower CHE levels (7449.00 ± 2286.00 vs 8687.00 ± 2270.00 U/L, t=10.30 6, *P <*0.001) (**Table 1**).

**Table 1:** Overall patients’ clinical characteristics and their comparisons between patients with and without significant hepatic histopathology.

Characteristics	Total (N = 1474)	No significant hepatic histopathology group (N = 764)	Significant hepatic histopathology group (N = 710)	*χ^2^*/t/z	*P*
**Male (%)**	56.20%	52.90%	59.90%	7.28	0.007
**Age (years)**	36.80 ± 10.60	35.70 ± 10.20	38.00 ± 10.80	-4.13	<0.001
**HBsAg (log_10_ IU/ml)**	3.30 ± 1.22	3.38 ± 1.25	3.20 ± 1.18	2.25	0.025
**Positive HBeAg (%)**	52.60%	46.70%	59.00%	22.3	<0.001
**HBV DNA (log_10_ IU/ml)**	5.30 ± 2.13	5.27 ± 2.27	5.32 ± 1.96	-0.44	0.663
**ALT (U/L)**	31.80 (21.20, 49.20)	26.80 (18.90, 40.70)	38.20 (25.70, 58.10)	-10.28	<0.001
**AST (U/L)**	27.80 (21.10, 40.20)	24.00 (19.30, 33.50)	32.80 (24.40, 48.90)	-11.69	<0.001
**TBIL (μmol/L)**	12.90 (10.00, 17.20)	12.40 (9.97, 16.40)	13.20 (10.10, 17.90)	-2.63	0.008
**ALB (g/L)**	45.20 ± 4.17	46.20 ± 3.58	44.10 ± 4.49	9.73	<0.001
**CHE (U/L)**	8087.00 ± 2359.00	8687.00 ± 2270.00	7449.00 ± 2286.00	10.36	<0.001
**PLT (10^9^/L)**	183.00 ± 56.90	199.00 ± 51.20	166.00 ± 57.90	11.52	<0.001
**PTA (%)**	89.20 ± 12.60	91.80 ± 11.80	86.90 ± 12.90	5.83	<0.001
**INR**	1.03 ± 0.09	1.03 ± 0.08	1.04 ± 0.10	-2.08	0.038

HBeAg, hepatitis B e antigen; HBsAg, hepatitis B surface antigen; HBV DNA, hepatitis B virus deoxyribonucleic acid; AST, aspartate aminotransferase; ALT, alanine aminotransferase; ALB, albumin; TBIL, total bilirubin; CHE, cholinesterase; PTA, prothrombin time activity; PLT, platelet; INR, international normalized ratio.

### Liver histopathology

Of the pathological diagnosis of liver tissue, the grade of liver inflammation: no G0, the proportion of G ≤ 1 was 61.0% (899 cases), the proportion of 1 < G ≤ 2 was 24.2% (356 cases), the proportion of 2 < G ≤ 3 was 13.7% (202 cases), and the proportion of 3 < G ≤ 4 was 1.2% (16 cases). Grading of liver fibrosis: S0 accounts for 0.8% (11 cases), S ≤ 1 accounts for 64.5% (951 cases), 1 < S ≤ 2 accounts for 20.6% (304 cases), 2 < S ≤ 3 accounts for 10.7% (157 cases), and 3 < S ≤ 4 accounts for 3.4% (50 cases) in **Table 2**.

**Table 2:** Histopathological grade distribution of the liver biopsy.

Classification	Degree of lesion	Grade	Number of people (%)
**G**	Insignificant	G0	0
		G ≤ 1	61.0%
	Significant	1<G ≤ 2	24.2%
		2<G ≤ 3	13.7%
		3<G ≤ 4	1.2%
**S**	Insignificant	S0	0.8%
		S ≤ 1	64.5%
	Significant	1<S ≤ 2	20.6%
		2<S ≤ 3	10.7%
		3<S ≤ 4	3.4%

G, inflammation grade; S, liver fibrosis stage.

### Comparison of hepatitis incidence under ALT threshold recommended by different guidelines

In the diagnostic criteria of 2018 AASLD chronic hepatitis B guidelines, the definition of normal ALT for men is ALT ≤ 35U/L, and that for women is ALT ≤ 25U/L. According to AASLD, 47.70% of patients in our study were normal and 52.30% were abnormal. Taking the normal range of ALT ≤ 40 U/L defined in 2019 Chinese Guidelines, 2019 EASL Guidelines, and 2016 APASL Guidelines as the normal range, 64.30% of patients in this study were normal patients and 35.70% were abnormal patients. Among the patients with normal ALT values, 36.40% and 40.40% of the patients under the two standards had significant hepatic histopathology, but without statistical difference (*χ^2 =^* 3.38, *P* =0.066). In patients with abnormal ALT values, 58.90% and 62.20% of the patients with the two criteria had significant hepatic histopathology, with no significant difference (*χ^2 =^* 2.28, *P* =0.131) (**Table 3**)

**Table 3:** Comparison of the occurrence of significant hepatic histopathology between normal and abnormal ALT groups under different guidelines.

	Guideline of AASLD	Guideline of China, EASL, APASL	*χ^2^* and *P*
**Normal value of ALT**	Male ≤ 35U/L, Female ≤ 25U/L	≤40 U/L	NA
**Percentage of ALT normal**	47.70% (703/1474)	64.30% (948/1474)	NA
**Percentage of ALT abnormalities**	52.30% (771/1474)	35.70% (526/1474)	NA
**Percentage of patients with normal ALT values but with significant hepatic histopathology**	36.40% (256/703)	40.40% (383/948)	*χ^2 =^* 3.38, *P* =0.066
**Percentage of patients with abnormal ALT values but with significant hepatic histopathology**	58.90% (454/771)	62.20% (327/526)	*χ^2 =^* 2.28, *P* =0.131

ALT, alanine aminotransferase; EASL, European Association for the Study of the Liver; APASL, Asian-Pacific Association for the Study of the Liver; AASLD, American Association for the Study of Liver Diseases.

### Analysis of factors associated with significant hepatic histopathology

In the analysis of factors associated with significant hepatic histopathology, by univariate logistic regression analysis, the results showed that significant hepatic histopathology were significantly associated with patients’ HBsAg (*P* = 0.025, OR = 0.883, 95% CI = 0.791 ~ 0.985), HBeAg positivity (*P* < 0.001, OR = 1.642, 95% CI = 1.335 ~ 2.018), and ALT levels (*P* < 0.001, OR=1.019, 95% CI=1.015~1.024) were significantly associated. By multivariate logistic regression analysis, the results showed that, HBsAg level (*P <*0.001, OR=0.665, 95% CI=0.577~0.767), HBeAg positivity (*P <*0.001, OR=2.131 95% CI=1.288~3.525), and ALT level (*P <*0.001, OR=1.014, 95% CI=1.002~1.025) were independent correlates of significant hepatic histopathology in chronic HBV infection (**Table 4**)

**Table 4:** Table of binary logistic regression analysis of overall patients regarding the occurrence of significant hepatic histopathology and cut off value for the variables.

Univariate binary logistic regression analysis				
	B	*P*	OR	95% CI
**HBsAg(log_10_ IU/ml)**	-0.13	0.025	0.883	0.791 ~ 0.985
**HBeAg-negative**	Ref	N/A	N/A	N/A
**HBeAg-positive**	0.50	<0.001	1.642	1.335 ~ 2.018
**HBV DNA(log_10_ IU/ml)**	0.01	0.663	1.011	0.962 ~ 1.060
**ALT(U/L)**	0.02	<0.001	1.019	1.015 ~ 1.024
**Multivariate binary logistic regression analysis**				
	**B**	***P***	**OR**	**95% CI**
**HBsAg(log_10_ IU/ml)**	-0.41	<0.001	0.665	0.577 ~ 0.767
**HBeAg-negative**	Ref	N/A	N/A	N/A
**HBeAg-positive**	0.81	<0.001	2.238	1.596 ~ 3.139
**ALT(U/L)**	0.02	<0.001	1.019	1.013 ~ 1.025
**Cut off value and AUC**				
	**Cut off**	**sensitivity**	**specificity**	**AUC**
**HBsAg(log_10_ IU/ml)**	3.85	42.60%	72.80%	0.56
**HBeAg**	0.50	59.00%	53.30%	0.56
**ALT(U/L)**	33.45	59.40%	65.40%	0.66

HBeAg, hepatitis B e antigen; HBsAg, hepatitis B surface antigen; HBV DNA, hepatitis B virus deoxyribonucleic acid; ALT, alanine aminotransferase; Ref, reference; N/A, not available; AUC, area under curve.

**Figure 2:**
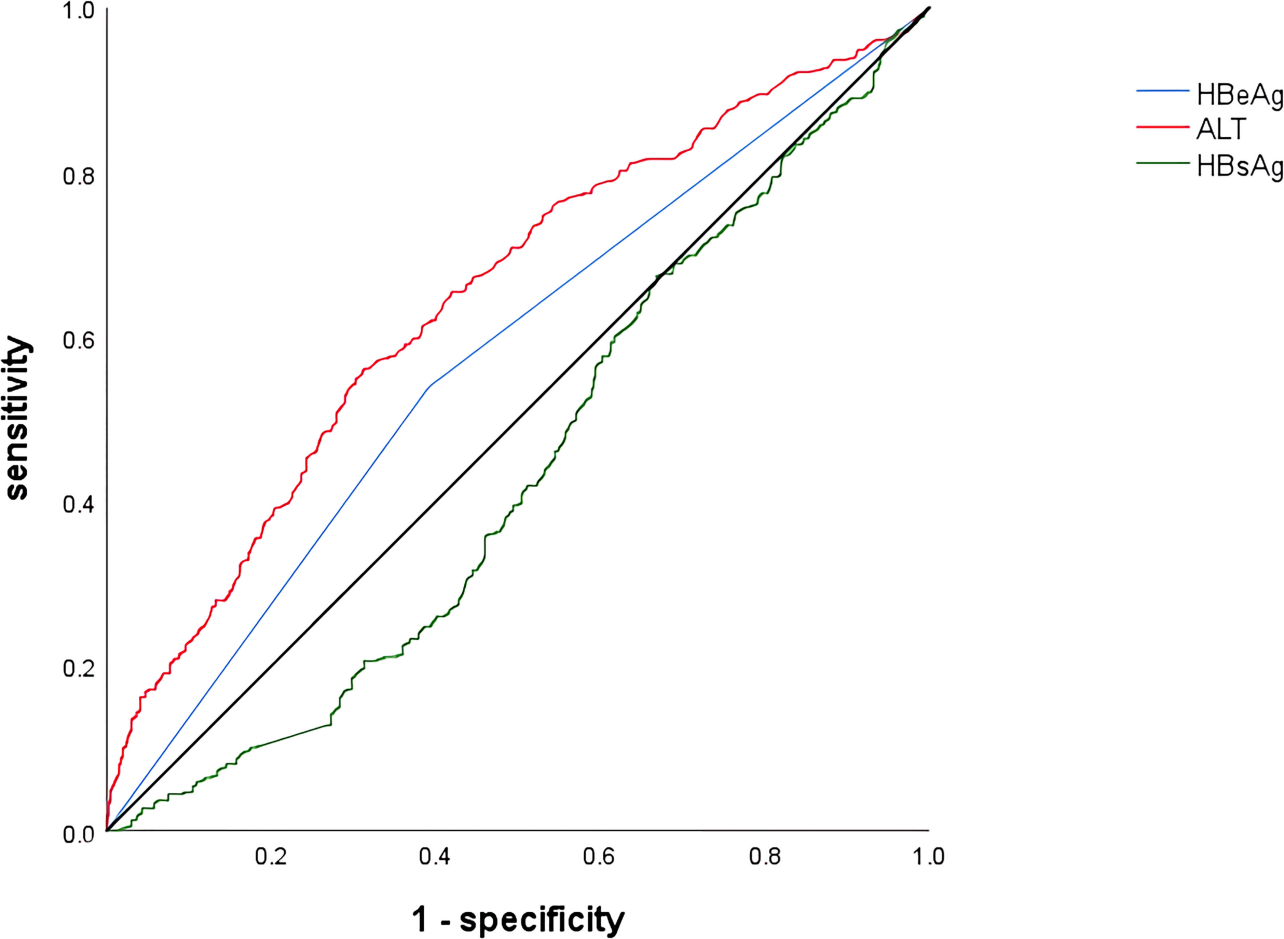
Receiver operating characteristic curve of variables associated with significant hepatic histopathology. HBeAg, hepatitis B e antigen; ALT, alanine aminotransferase; HBsAg, hepatitis B surface antigen.

The cutoff values were calculated from the receiver operating characteristic curve (ROC) and the Jorden index, and the results showed that HBsAg (3.85 log10 IU/ml, sensitivity 42.60%, specificity 72.80%, AUC=0.56) and ALT (33.45 U/L, sensitivity 59.40%, specificity 65.40%, AUC=0.66) (**Table 4**; **Figure 2**)

### Correlation analysis of ALT with G and S in patients with significant hepatic histopathology

In patients with significant hepatic histopathology, grade of inflammation G gradually increased with increasing ALT levels (r =0.194, *P <*0.001), while liver fibrosis stage S gradually decreased (r =-0.066, *P* =0.021).

## Discussion

As we know, G≥2 means the presence of significant inflammation and S≥2 means the presence of significant fibrosis. In our study, we found that 5.3% (78) patients had pathological findings suggestive of G or S between 1-2 by analyzing the liver biopsy results of all patients. We defined G>1 and/or S>1 as the presence of significant hepatic histopathology in order to cover those patients between 1-2, because significant hepatic histopathology were truly absent in patients with G ≤ 1 and S ≤ 1. We believed that patients between 1-2 were in the early stages of inflammation or fibrosis and that disease progression would occur without intervention.

HBV is a non-cytopathogenic hepatophilic virus, and it is currently believed that it does not cause damage to liver cells per se. Liver inflammation occurs when HBV induces an immune response in the host, causing hepatocyte necrosis *via* immune damage, which may lead to cirrhosis or even liver cancer if left untreated. The key to whether chronic HBV-infected patients need to initiate antiviral therapy is the presence of liver inflammation, with inflammation requiring treatment. HBV stimulates the body’s immune response, thereby causing hepatocyte necrosis, liver tissue inflammation, and fibroplasia through immune cells and secreted cytokines, which results in the occurrence of chronic hepatitis B. However, viral antigens inhibit the effective clearance of viruses and infected liver cells by virus-specific immune cells, making the disease persistent and preventing spontaneous recovery from infection. Therefore, antiviral therapy is the most important way to stop the progression of liver disease.

The key to the need for antiviral therapy is the presence of liver inflammation, then it is very important to accurately identify the presence of liver inflammation. Clinical indicators in patients with HBeAg positive chronic infection stage are characterized by high HBV DNA load, HBeAg positivity and normal ALT (2). Truly immune-tolerant patients usually meet these criteria, and most scholars now believe that patients in the immune-tolerant phase have no or only mild liver inflammation and therefore do not require antiviral therapy (7–9). ALT has the most sensitive reaction of hepatocyte inflammation and necrosis, and a small amount of hepatocyte necrosis can cause an increase in ALT, but not all patients with hepatic histological lesions exhibit elevated ALT, meaning that even if the criteria for the immune tolerance period are met, significant hepatic histopathology may still be present.

The latest version of the Hepatitis B guidelines, including China, EASL and APASL, all define a normal ALT value as less than 40 U/L (2, 3, 5). However, many scholars have studied the liver pathology findings of chronic HBV-infected patients with normal ALT and found that different proportions of this group of patients already had hepatitis (10–12), that is, the criteria for antiviral treatment were met. In our previous study, we found that 20.2% of patients with chronic HBV infection who met the conditions of HBV DNA>2×10 (7) IU/ml, HBeAg positivity, and ALT ≤ 40 U/L had liver puncture biopsies result suggesting liver inflammation (13).

In addition, with the growth of age, more and more patients in the immune tolerance phase will break tolerance to hepatitis and enter the immune clearance phase, and a previous study found that the median age of immune tolerance breakage was 30.7 years (14). In view of the above characteristics, the need to lower the ALT threshold for initiating antiviral therapy in patients who were chronic infected with HBV has been widely discussed by scholars in recent years. Most scholars believe that lowering the ALT threshold for antiviral therapy can effectively improve the diagnosis rate as well as the treatment rate of hepatitis B, so that more patients with hepatitis B can benefit from it (4, 15).

In this study, the histopathological findings of the liver of patients with ALT ≤ 40 U/L were analyzed separately without considering other clinical indicators, and the results showed that 40.40% had significant hepatic histopathology. We compared the diagnostic criteria of ALT ≤ 35 U/L in men and ALT ≤ 25 U/L in women with the ALT ≤ 40 U/L recommended by the 2019 edition of the Chinese guidelines for chronic hepatitis B, the 2019 edition of the EASL guidelines and the 2016 edition of the APASL guidelines by referring to the ALT diagnostic criteria of the 2018 edition of the AASLD guidelines for chronic hepatitis B. The results showed that 36.40% and 40.40% of patients with normal ALT under the AASLD criteria and the remaining guideline criteria had significant hepatic histopathology, respectively, but with no statistically significant differences. In contrast, among patients with abnormal ALT values, 58.90% and 62.20% of patients with significant hepatic histopathology by AASLD criteria and Chinese guideline criteria, respectively, and the difference was also not significant.

After studying the role of ALT to predict liver fibrosis, Korean scholars found that there was a positive correlation between ALT levels derived from the liver and liver fibrosis stage, but no correlation was found between overall serum ALT levels and fibrosis stage (16). In our study, we found that ALT levels were positively correlated with G, but inversely correlated with S. Combined with the studies of previous scholars, we believed that the reason for the difference might be due to variances in scoring criteria and ALT measurement methods. The correlation coefficient between ALT and S was only -0.066, and the clinical significance of this result was too small to be used for clinical guidance. However, we can also learn from this that the relationship between ALT and G is stronger than the relationship between ALT and S, which is in accordance with our knowledge.

It is not enough to rely on ALT alone as an indicator to assess the necessity of HBV therapy, as hepatitis attacks may be accompanied by changes in several clinical indicators, such as HBsAg, HBV DNA, alpha fetoprotein (AFP), etc (17, 18). Also, when evaluating a patient, some demographic characteristics such as age and gender are factors that we must consider (19). The best strategy is to evaluate the need to initiate antiviral therapy in patients with CHB by combining multiple indicators, as our current treatment goals for CHB have changed, and clinical cure based on delaying disease progression is the best (20, 21). Similar conclusions were found in our study. After analyzing the risk factors associated with significant hepatic histopathology, we found that in addition to ALT, HBsAg levels and HBeAg status were also associated with significant hepatic histopathology. Although the diagnostic ability of each indicator alone is unsatisfactory, the combination of several indicators to determine the disease progression status in clinical work can make the diagnosis more reliable.

We found that 48.17% of untreated patients had significant hepatic histopathology by enrolling a large sample size of HBV-infected patients, that means a significant proportion of patients requiring treatment existed under the normal ALT criteria of different guidelines, which suggested that lowering the ALT threshold for initiating treatment in CHB patients was important and necessary to improve the prognosis and reduce the burden of disease. At the same time, there were some shortcomings in this study, as we were not able to calculate the optimal threshold for initiation of treatment, and the enrolled patients did not cover multiple ethnic groups. However, these problems need to be solved by designing a more elaborate study protocol.

## Data availability statement

The raw data supporting the conclusions of this article will be made available by the authors, without undue reservation.

## Author contributions

ML and YX contributed to the study design. ZZ, HH, XB, YLi, LY, SW contributed to the data analysis. GS, MC, YLu, YG, RL, MX, XC, LH, LZ contributed to the recruitment, enrolment, and assessment of participants, as well as following up with the patients. TJ, WD, FS, HL contributed to data collection. ZZ and ML wrote the first draft of the manuscript. YX revised the manuscript and is the guarantor of the article. All authors contributed to the article and approved the submitted version.

## Funding

The Digestive Medical Coordinated Development Center of Beijing Hospitals Authority (XXZ0302 and XXT28). Project supported by Beijing science and technology commission (Z211100002921059). High-level Public Health Technical Personnel Training Program of Beijing Municipal Health Commission (2022-3-050). National Science and Technology Major Project of China (2017ZX10201201-001-006, 2017ZX10201201-002-006, 2018ZX10715-005-003-005). The capital health research and development of special (2022-1-2172). Beijing Hospitals Authority Clinical Medicine Development of special funding support (XMLX 202127). National Key R&D Program of China (2022YFC2603500)

## Conflict of interest

The authors declare that the research was conducted in the absence of any commercial or financial relationships that could be constructed as a potential conflict of interest.

The reviewer ZC declared a shared parent affiliation with the authors to the handling editor at the time of review.

## Publisher’s note

All claims expressed in this article are solely those of the authors and do not necessarily represent those of their affiliated organizations, or those of the publisher, the editors and the reviewers. Any product that may be evaluated in this article, or claim that may be made by its manufacturer, is not guaranteed or endorsed by the publisher.
